# Unlocking Potential of *Potentilla erecta*: Development and Efficacy Evaluation of Oral Mucoadhesive Gel for Oral Ulcers

**DOI:** 10.3390/gels12070616

**Published:** 2026-07-09

**Authors:** Tamara Rudic, Jovana Bradic, Jasmina Sretenovic, Aleksandar Kocovic, Miona Vuletic, Suzana Zivanovic, Irena Petrusic, Vladimir Jakovljevic, Aleksandra Stojanovic

**Affiliations:** 1 Center of Excellence for the Study of Redox Balance in Cardiovascular and Metabolic Disorders, Faculty of Medical Sciences, University of Kragujevac, 34000 Kragujevac, Serbiadrvladakgbg@yahoo.com (V.J.); vranicaleksandra90@gmail.com (A.S.); 2 Department of Pharmacy, Faculty of Medical Sciences, University of Kragujevac, 34000 Kragujevac, Serbia; 3 Department of Physiology, Faculty of Medical Sciences, University of Kragujevac, 34000 Kragujevac, Serbia; 4 Department of Dentistry, Faculty of Medical Sciences, University of Kragujevac, 34000 Kragujevac, Serbia; 5 Faculty of Management Herceg Novi, University Adriatic, 85348 Herceg Novi, Montenegro; 6 Department of Pathophysiology, 1st Moscow State Medical University IM Sechenov, 119991 Moscow, Russia

**Keywords:** *Potentilla erecta*, oral ulceration, hydrogel, oxidative stress, rats

## Abstract

Oral ulcerations are complex pathological lesions with multifactorial etiology and diverse clinical manifestations. Current treatment options are mostly symptomatic with a different adverse effect. Therefore, this study aimed to develop a mucoadhesive oral gel containing *Potentilla erecta* L. ethanol extract (PEOG) and evaluate its healing effects in a rat model of oral ulceration. Dried rhizomes of *Potentilla erecta* were extracted with 70% ethanol using ultrasonic extraction, followed by low-pressure evaporation. The extract was incorporated into a gel base composed of poloxamer 407 and carbomer 934. Rheological characterization was performed to assess the viscoelastic and flow properties of the formulation. Therapeutic efficacy was evaluated through macroscopic assessment of ulcer healing, histopathological analysis, and determination of systemic oxidative stress biomarkers. Animals were assigned to three groups: untreated control, gel base (GB), and PEOG-treated. Rats were sacrificed on days 0, 3, 6, and 10 for blood and tissue sampling. PEOG treatment significantly accelerated ulcer healing, resulting in a marked reduction in ulcer size compared with controls. Histopathological findings indicated enhanced collagen deposition, while biochemical analyses suggested attenuation of oxidative stress. These results demonstrate that PEOG possesses considerable ulcer-healing potential and may represent a promising mucoadhesive formulation for the treatment of oral ulcerations.

## 1. Introduction

Oral ulcerations represent a heterogeneous and complex pathological entity characterized by diverse etiology, clinical manifestations, and multifactorial pathogenesis. Although some cases may be explained by local irritants, such as mechanical factors (e.g., sharp edges of retained roots or crowns), physical factors (thermal injuries), or chemical agents (strong acids and bases), most arise as a consequence of the interaction between local and systemic causes. The diagnosis and treatment of different forms of these lesions require professional expertise and a thorough clinical approach. In addition to common forms, there are also rare types in which establishing an accurate diagnosis can often be challenging at first presentation [1,2].

These lesions are characterized by damage to the oral epithelium, with partial or complete loss of connective tissue, resulting in a characteristic crater-like appearance. They may occur as a consequence of trauma, infection, or allergic reactions and are frequently associated with dermatological, autoimmune, and neoplastic diseases, as well as inflammatory bowel diseases [2,3]. For example, recurrent aphthous ulcerations may be associated with immune dysregulation, genetic predisposition, nutritional deficiencies, local trauma, or psychological factors such as stress and anxiety [4,5].

From a morphological perspective, some ulcerations exhibit distinctive clinical features. Recurrent aphthous ulcerations are usually well circumscribed, round or oval, with a whitish-yellow pseudomembrane surrounded by an erythematous halo. Traumatic ulcerations are easily recognized by their location and shape corresponding to the causative factor. Tuberculous ulcerations are stellate in shape, with undermined edges and clearly defined margins [6]. However, many ulcerations lack a typical appearance, making diagnosis more difficult. While some can be readily identified based on clinical presentation and medical history, others require a detailed and comprehensive evaluation due to unclear symptoms and a complex medical background [1,2,7]. Ulcerations associated with medications, autoimmune diseases, and infectious conditions represent a particular diagnostic challenge [8].

Therapy may be local or systemic, with the aim of providing analgesia, promoting adequate ulcer healing, controlling pain, and preventing recurrence. Local therapy includes the use of anesthetics, antiseptics, analgesics, corticosteroids, antimicrobial agents, cyclosporine, and retinoids. Systemic therapy includes the administration of steroids, dapsone, colchicine, pentoxifylline, thalidomide, and similar agents, representing the second step in treatment. The use of immunomodulators or systemic immunosuppressants requires careful monitoring and is associated with numerous adverse effects. Therefore, there is a need to explore new and safer therapeutic approaches [9].

*Potentilla erecta* L. (*P. erecta*) has emerged as a valuable source of compounds with significant anti-inflammatory properties that may be used as ingredients in the development of effective dermocosmetic products, acting through the inhibition of interleukin-6 and prostaglandin E2. In this way, it demonstrates an effect comparable to hydrocortisone and shows potential for application in the inflammatory skin diseases treatment. This opens the possibility for the use of *P. erecta* extract as the bioactive component in the development of drug delivery systems for the treatment of inflammatory skin diseases [10], and potentially for the treatment of oral ulcerations. Therefore, the aim of our study was to prepare a mucoadhesive oral gel based on *Potentilla erecta* L. ethanol extract (PEOG) and reveal its healing effects in the model of oral ulcerations in rats.

## 2. Results and Discussion

### 2.1. Physicochemical Characterization of PEOG

#### 2.1.1. Physical Stability and Sensory Characteristics

The organoleptic properties of the PEOG, including color, odor, consistency, and texture, were monitored under different storage conditions for a duration of three months (Table 1). Throughout this period, the gel retained a uniform appearance without signs of phase separation, or change in odor, indicating good formulation stability. Importantly, the consistency of the gel remained unchanged, confirming that no degradation or destabilization occurred during storage. The absence of alterations in organoleptic properties indicates that formulated gel remained stable during the follow-up period. Such stability is likely attributed to the synergistic role of polyacrylic acid derivatives and poloxamer 407 in maintaining a coherent gel matrix, which has previously been reported to enhance the physicochemical robustness of hydrogel systems [11]. These findings demonstrate that the PEOG is not only chemically active but also physically stable, which is essential for ensuring reproducible therapeutic performance and patient acceptability during prolonged use.

#### 2.1.2. pH Measurements

The pH of the PEOG was monitored over 90 days at two storage temperatures (25 ± 2 °C and 4 ± 2 °C) to assess its stability for oral mucosal application (Table 2). On day 1, the gel exhibited pH values of 5.75 ± 0.11 at 25 °C and 5.79 ± 0.12 at 4 °C, which slightly increased to 5.91 ± 0.14 and 5.88 ± 0.16, respectively, by day 90. Furthermore, the observed pH values fall within the physiologically acceptable range for oral tissues (5.5–7.9), confirming the gel’s suitability for safe topical use in the mouth [12]. The formulation maintained chemical stability and compatibility with the oral environment. Monitoring pH is critical in assessing formulation stability, as substantial deviations may suggest degradation or incompatibility of components [13,14]. However, in this case, the observed variations were minimal and are unlikely to affect safety or efficacy. Furthermore, the combination of ellagic acid-rich extract and the polymeric gel matrix did not cause any notable destabilization, supporting that the PEOG is stable and suitable for topical application on oral mucosa.

#### 2.1.3. Electrical Conductivity Measurements

Electrical conductivity in hydrogels arises primarily from the movement of ions and electrons, making it a useful indicator of gel uniformity, stability of the polymer network and structural stability. To evaluate the physicochemical behavior of PEOG hydrogel, conductivity was measured over 90 days at 25 ± 2 °C and 4 ± 2 °C (Table 3). Initially (day 1), both temperatures showed similar values of 50.7 ± 0.50 µS/cm, reflecting a well-dispersed ionic environment within the gel matrix. After 90 days, the sample stored at 25 °C showed a modest decrease from 50.7 ± 0.50 µS/cm to 46.1 ± 0.58 µS/cm, possibly due to minor water loss or relaxation of the polymer network, whereas the sample stored at 4 °C exhibited a marked increase to 68.3 ± 0.60 µS/cm, likely related to enhanced ion mobility or better solubilization of ellagic acid and other extract constituents. Both changes were statistically significant, with the 4 °C value also differing significantly from the 25 °C sample.

#### 2.1.4. Centrifugation Test

Upon subjecting the PEOG to centrifugation, no visible phase separation, sedimentation, or breakdown of the gel components was observed, indicating that the formulation maintained its structural integrity and stability.

#### 2.1.5. Rheological Characterization

**Figure 1:**
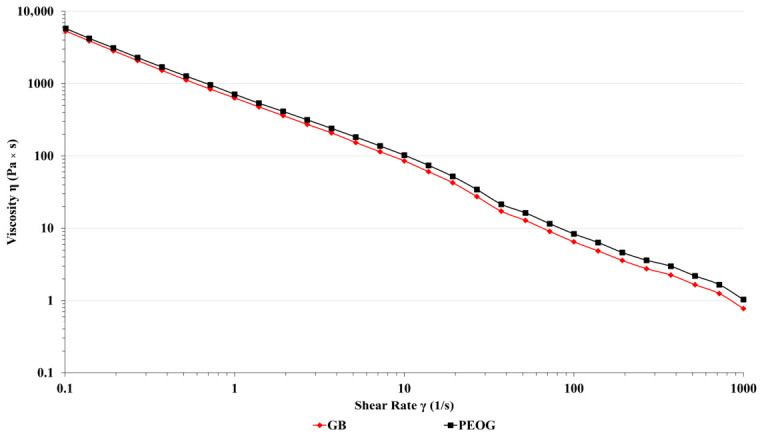
Viscosity curve of PEOG and GB 48 h after preparation.

Rheological characterization of the developed PEOG oral gel was performed at 25 °C, and the obtained viscosity curve is shown in Figure 1. The flow behavior analysis revealed that the formulation follows a non-Newtonian, pseudoplastic pattern, whereby an increase in shear rate leads to a progressive reduction in viscosity. Such behavior is particularly advantageous for gels designed for topical application onto oral mucosal surfaces, since the formulation becomes more fluid during spreading and regains its consistency once applied, allowing it to remain at the target site for an extended period. The importance of shear-thinning properties in mucoadhesive systems has been well established in the scientific literature. Mandal et al. pointed out that polymer-based delivery systems intended for mucosal surfaces must undergo viscosity reduction under mechanical stress to allow comfortable administration, while preserving gel structure under resting conditions to support localized therapeutic action [15]. The clinical relevance of our findings is further supported by the work of Pecora et al., who established that the capacity of a mucoadhesive oral products to form a stable film and withstand the mechanical forces of saliva and tongue movement is closely tied to its viscoelastic characteristics, a factor of particular significance when treating painful mucosal lesions such as aphthous ulcers [16]. Taken together, the rheological profile of the formulation developed in this study aligns well with the criteria outlined in the current literature for mucoadhesive oral gels, indicating its potential suitability for clinical use.

#### 2.1.6. Swelling Index

The swelling behavior of the PEOG immediately after preparation and after 90 days of storage at 4 ± 2 °C and 25 ± 2 °C is summarized in Table 4. On day 1, the gel exhibited a progressive increase in swelling over time, with the highest values observed at the 3 h measurement (133 ± 0.12). After 90 days, the formulation stored at 25 °C showed the greatest swelling at 3 h (110 ± 0.20), whereas the sample stored at 4 °C displayed a gradual decrease in swelling over the same period, reaching 72 ± 0.17 at 3 h. Overall, these results indicate that the PEOG maintains its swelling capacity over time, with storage temperature influencing the extent of water uptake, which is important for its sustained release and mucoadhesive performance on the oral mucosa.

### 2.2. Antioxidative Activity

The total phenolic content (TPC) of PE was determined to be 112.32 ± 6.41 mg GAE/g dry extract. In the FRAP assay, PE exhibited a ferric-reducing capacity of 1.26 ± 0.21 µM Fe^2+^ equivalents, whereas ascorbic acid showed a markedly higher reducing power (2.15 ± 0.21 µM Fe^2+^ equivalents).

The antioxidant activity was evaluated using DPPH and ABTS radical-scavenging assays, is presented in Table 5. Significant differences in antioxidant activity were observed among the tested samples in both assays. *P. erecta* exhibited significantly higher IC_50_ values than all reference antioxidants in both assays, indicating weaker radical-scavenging activity. In contrast, no statistically significant differences were observed among the standard antioxidants (ascorbic acid, BHA, and Trolox).

The present study investigated the phytochemical composition and antioxidant potential of *P. erecta* rhizome extract, with the aim of establishing a rational basis for its incorporation into a mucoadhesive oral gel intended for the treatment of recurrent aphthous stomatitis. Considering the well-documented role of oxidative stress in the pathogenesis of oral ulcerations, characterized by impaired enzymatic antioxidant defences and elevated levels of reactive oxygen species in both serum and oral tissues of affected patients [17,18] the selection of a plant extract with verified antioxidant capacity represents a sound and evidence-based pharmacological strategy.

Phytochemical characterization of the investigated *P. erecta* extract revealed a total phenolic content of 112.32 ± 6.41 mg GAE/g dry extract, as determined by the Folin–Ciocalteu method. This value is consistent with previously reported data for *P. erecta* rhizome extracts obtained under comparable extraction conditions, where total phenolic contents have been reported to vary considerably depending on geographical origin, harvest period, and solvent polarity [19,20]. The relatively high phenolic content observed in our extract is in agreement with the well-characterized phytochemical profile of *P. erecta* extract, which is particularly rich in hydrolysable tannins, most notably agrimoniin, pedunculagin, and ellagic acid derivatives, as well as condensed tannins and phenolic acids [21,22]. These compounds are generally considered the primary contributors to the antioxidant activity of this plant.

The antioxidant capacity of *P. erecta* extract was evaluated using three complementary in vitro assays: DPPH radical scavenging, ABTS radical cation decolorization, and ferric-reducing antioxidant power (FRAP). The extract exhibited IC_50_ values of 58.94 ± 5.09 μg/mL and 68.67 ± 7.04 μg/mL in the DPPH and ABTS assays, respectively, and a ferric-reducing capacity of 1.26 ± 0.21 μM Fe^2+^ equivalents in the FRAP assay. In both radical-scavenging assays, the extract demonstrated significantly higher IC_50_ values compared with all reference antioxidants, ascorbic acid, BHA, and Trolox, indicating weaker radical-scavenging activity relative to pure standards (Tukey post hoc, *p* < 0.001). No statistically significant differences were detected among the reference antioxidants themselves. Similarly, in the FRAP assay, ascorbic acid exhibited a markedly higher reducing capacity (2.15 ± 0.21 μM Fe^2+^eq.) compared to *P. erecta* extract.

Although the radical-scavenging activity of *P. erecta* was weaker than that of the pure reference compounds, this finding is expected and does not diminish the biological relevance of the extract. The antioxidant effects of plant extracts arise from synergistic interactions among multiple bioactive constituents rather than from a single highly potent molecule, and it is generally accepted that combinations of phenolic antioxidants exert better overall activity than isolated compounds [23,24]. Moreover, moderate in vitro IC_50_ values do not preclude significant efficacy in complex biological systems. A direct comparison of crude extracts with pure synthetic antioxidants does not accurately reflect the multifaceted mechanisms through which plant phenolics exert their effects in vivo, which include, beyond direct radical scavenging, modulation of endogenous antioxidant enzyme activity, metal ion chelation, and inhibition of pro-oxidant enzymes [24]. This is particularly relevant in the context of oral ulcerations, where the therapeutic target is not simply the neutralization of free radicals, but the restoration of the overall redox balance through augmentation of SOD, CAT, and GSH activity, mechanisms that are inadequately captured by cell-free radical-scavenging assays alone.

The antioxidant activity observed in our study is in line with previously published data for *P. erecta* extracts. Kaltalioglu et al. reported IC_50_ values in the DPPH assay of approximately 35–70 μg/mL for ethanolic rhizome extracts of *P. erecta* [25], and comparable values have been reported by Tomczyk and Latté across various Potentilla species and extract types [21] supporting the reproducibility of our findings. The somewhat lower TPC value obtained in our study relative to some published reports may reflect differences in the degree of extraction, drying procedures, or the specific chemotype of the plant material used, all of which are known to substantially influence the yield of phenolic compounds [19,20].

The use of three independent assays based on different mechanistic principles, hydrogen atom transfer (DPPH), electron transfer (ABTS), and reduction of Fe^3+^ (FRAP), provides a more comprehensive picture of the antioxidant profile of the extract than any single method would allow, and is consistent with current methodological recommendations [24]. The convergent results obtained across these three assays reinforce the conclusion that PE possesses genuine antioxidant potential. The moderate reducing capacity observed in the FRAP assay is consistent with the presence of ellagic acid and ellagic acid derivatives, which have been identified as major constituents in our extract (14.99 ± 1.2 μg/mg and 23.25 ± 1.11 μg/mg, respectively), and which are known to exhibit Fe^3+^-reducing activity through electron-donating mechanisms [26]. Epicatechin (7.8 ± 0.3 μg/mg), also detected in the extract, is a well-characterized antioxidant catechin known to exert its effects through free radical scavenging, singlet oxygen quenching, and transition metal chelation [27], and its presence likely contributes to the activity observed across all three assays.

From a pathophysiological standpoint, the antioxidant properties of *P. erecta* extract are particularly relevant considering the established role of oxidative stress in oral ulcerations. Previous studies have demonstrated that patients with aphthous stomatitis exhibit significantly elevated levels of lipid peroxidation products and reactive oxygen species, alongside reduced activities of SOD, CAT, and glutathione peroxidase in both serum and saliva [17,18,28]. This oxidative imbalance is thought to perpetuate mucosal damage and impair the normal healing process. The antioxidant constituents of *P. erecta*, particularly ellagic acid and its derivatives, have been shown to activate the Nrf2/ARE signaling pathway, thereby upregulating endogenous antioxidant defenses and reducing oxidative tissue damage [29,30]. These mechanisms are consistent with the accelerated ulcer healing and improved redox parameters observed in vivo in the PEOG-treated group, as presented in the subsequent sections of this study.

Taken together, the phytochemical and antioxidant data presented in this section establish that PE represents a phenolic-rich extract with verifiable radical-scavenging and reducing capacity. These properties, arising from the combined activity of ellagic acid, ellagic acid derivatives, and epicatechin, provide a sound mechanistic rationale for the incorporation of this extract into the mucoadhesive oral gel formulation and support its further evaluation in the experimental model of oral ulceration.

### 2.3. Ulcer Healing Rate (%)

**Figure 2:**
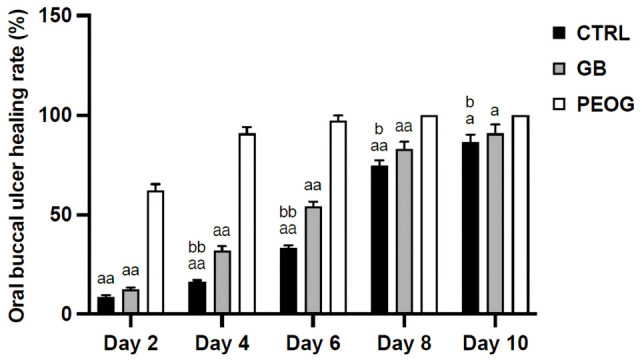
Oral buccal ulcer healing rate (%) at multiple time points (days 2, 4, 6, 8, and 10) across different treatment groups. CTRL—control, untreated rats; GB—rats treated with gel base; PEOG—rats treated with *P. erecta* oral gel. a—compared to PEOG; b—compared to GB; a and b—*p* < 0.05; aa, bb—*p* < 0.01.

**Figure 3:**
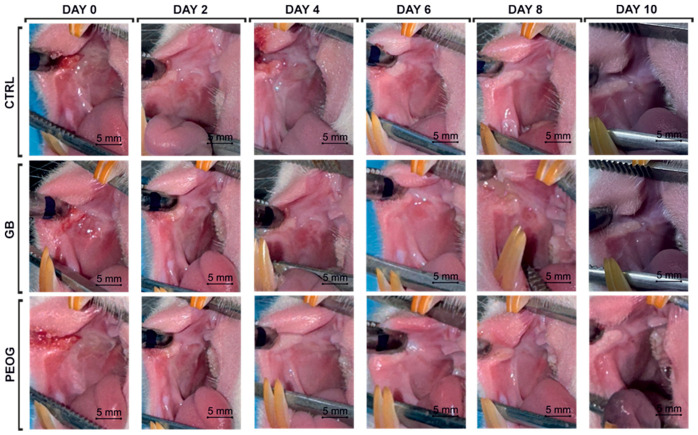
Macroscopic changes in ulcer size at multiple time points (days 0, 2, 4, 6, 8, and 10) across different treatment groups. CTRL—control, untreated rats; GB—rats treated with gel base; PEOG—rats treated with *P. erecta* oral gel.

The healing potential of the PEOG was assessed over a 10-day period by evaluating ulcer contraction every second day. As shown in Figure 2 and Figure 3, the PEOG treatment demonstrated a significantly accelerated healing effect compared to the GB and control CTRL groups. A rapid reduction in ulcer size was observed early in the treatment, with PEOG achieving 62.0 ± 3.4% contraction by day 2, roughly five times greater than CTRL and substantially higher than GB. By Day 4, the PEOG group reached 90.8 ± 3.2%, clearly outpacing GB and CTRL. A strong upward trend continued, and nearly complete ulcer closure (97.1 ± 2.7%) was evident in PEOG-treated rats by day 6, while the GB and CTRL groups showed 54.3 ± 2.4% and 33.5 ± 1.2%, respectively. Notably, while PEOG achieved full epithelialization, by day 8, whereas the GB and CTRL groups reached only 90.8 ± 4.5% and 86.3 ± 3.98% by day 10. Overall, the PEOG formulation enhanced healing by approximately two- to four-fold across key evaluation days, highlighting its strong therapeutic potential in treating oral mucosal injuries. The significantly enhanced healing observed with PEOG can be attributed to the potent bioactive composition of *P. erecta* extract, notably rich in ellagic acid (14.99 ± 1.2 µg/mg), ellagic acid derivatives (23.25 ± 1.11 µg/mg), and epicatechin (7.8 ± 0.3 µg/mg), which are known for their strong antioxidant and anti-inflammatory activities. In our previous study, we investigated *P. erecta* extract incorporated into gel and confirmed its ability to reduce inflammation through both in silico and in vivo analyses. These earlier findings support the present results, suggesting that the extract, when delivered via a mucoadhesive gel, creates a favorable microenvironment that promotes faster epithelialization and more efficient ulcer healing [14].

Wound healing is a multifaceted pathophysiological process that involves the coordination of various cellular and biochemical events. It is an intricate mechanism encompassing inflammation, re-epithelialization, angiogenesis, the formation of granulation tissue, and collagen deposition [31]. Previous studies indicated that catechins, such as epicatechin gallate, can significantly improve the quality of wound healing and scar formation [32].

### 2.4. Histological Analysis

Histological assessment is essential for elucidating the stages and mechanisms of oral wound healing, providing a basis for evaluating the therapeutic efficacy of PEOG and for monitoring the time-dependent effects throughout the 10-day protocol. Table 6 presents the histological scoring results for buccal ulcer tissues collected on days 0, 3, 6, and 10.

**Figure 4:**
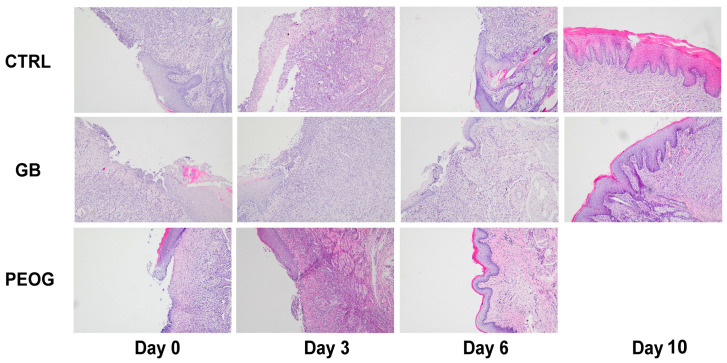
Microphotographs of H/E staining of the oral ulcer on days 0, 3, 6, and 10 (magnification 100×). CTRL—control, untreated rats; GB—rats treated with gel base; PEOG—rats treated with *P. erecta* oral gel.

Histopathological analysis of the rat buccal mucosa performed on day 3 demonstrated epithelial necrosis, partial ulceration, prominent subepithelial inflammatory infiltration predominantly composed of neutrophils, and vascular dilation across all experimental groups. Histological examination performed on day 6 revealed a moderate chronic inflammatory response, characterized by fibroblast proliferation and organized collagen fiber deposition in both the GB and CTRL groups. The PEOG-treated group showed signs of connective tissue remodeling and full ulcer resolution. By day 10, connective tissue formation indicative of healing was evident in both the CTRL and GB groups. Healing scores were nearly matching those observed in the PEOG group (Figure 4). The potential therapeutic effects of PEOG in the treatment of oral ulcers were confirmed in the present study. Specifically, animals treated with this gel exhibited complete ulcer healing after six days of treatment, whereas complete recovery was not observed in the CTRL and GB groups. Several mechanisms may account for these beneficial effects. As is well established, oxidative stress plays a crucial role in the pathogenesis of recurrent aphthous stomatitis (RAS). Excessive production of reactive oxygen species can lead to cellular damage, lipid peroxidation, DNA injury, prolonged inflammation, and delayed re-epithelialization. Since our study demonstrated a reduction in pro-oxidant markers accompanied by an increase in antioxidant defenses, we believe that the antioxidant activity of PEOG represents one of the key mechanisms underlying its therapeutic efficacy. Furthermore, oxidative stress and inflammation are closely interconnected processes. In the present study, a reduced infiltration of inflammatory cells was observed in the treated animals, supporting the anti-inflammatory potential of PEOG. These findings are consistent with previous reports showing that *P. erecta* extract decreases the levels of pro-inflammatory mediators, including IL-6 and PGE_2_, and exerts anti-inflammatory effects through inhibition of the NF-κB signaling pathway. In addition to its anti-inflammatory activity, *P. erecta* has also been reported to exert vasoconstrictive effects by inhibiting endothelial nitric oxide synthase (eNOS) activity and scavenging nitric oxide (NO) radicals [33]. These mechanisms may further contribute to the healing process by reducing local inflammation and tissue damage.

The positive histological effects observed in our study can be attributed, at least in part, to ellagic acid, one of the most dominant bioactive compounds in the mucoadhesive PEOG. Consistent with previous findings, systemic administration of ellagic acid has been shown to accelerate socket healing after tooth extraction in both healthy and diabetic rat models, through improvement in antioxidant status and histological parameters [34]. Unlike earlier studies where ellagic acid was administered systemically in a tooth extraction model, our approach employed a topical, sustained-release formulation, suggesting that localized delivery of ellagic acid can effectively promote tissue repair. To the best of our knowledge, no studies have directly addressed histological outcomes of ellagic acid-based extract incorporated in gel in oral ulcer healing, highlighting the novelty of our results.

### 2.5. Systemic Oxidative Stress Markers

**Figure 5:**
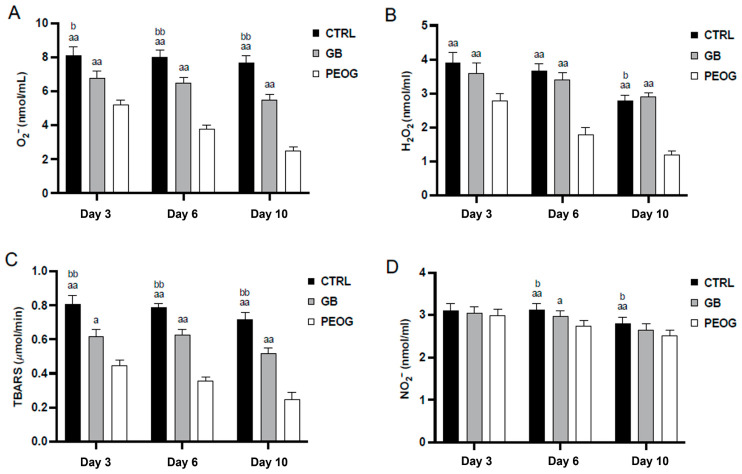
Values of prooxidative parameters in the plasma samples. (**A**)—superoxide anion radical (O_2_^.−^), (**B**)—hydrogen peroxide (H_2_O_2_); (**C**)—lipid peroxidation index (TBARS); (**D**)—nitrites (NO^2−^). CTRL—control, untreated rats; GB—rats treated with gel base; PEOG—rats treated with *P. erecta* oral gel. a—compared to PEOG; b—compared to GB; a and b—*p* < 0.05; aa and bb—*p* < 0.01.

Significant changes in oxidative stress markers were observed across all experimental groups over time, with the PEOG-treated group consistently demonstrating the strongest reduction in pro-oxidant levels. In the PEOG group, both H_2_O_2_ and O_2_^−^ levels decreased progressively from day 3 to day 10, reflecting a robust antioxidant effect. H_2_O_2_ concentrations dropped from 2.8 ± 0.2 nmol/mL on day 3 to 1.2 ± 0.1 nmol/mL on day 10, while O_2_^−^ levels decreased from 5.2 ± 0.3 to 2.5 ± 0.2 nmol/mL in the same period. In contrast, the GB and CTRL groups maintained elevated levels of both markers compared to PEOG, with slight decline in H_2_O_2_ values and consistently high levels of O_2_^−^ in the CTRL group during the follow-up period. The GB group showed intermediate values but remained significantly higher than PEOG throughout the study. Notably, on day 10, O_2_^−^ in the CTRL group was more than three times higher than in PEOG (Figure 5A), and H_2_O_2_ levels were more than double (Figure 5B). Additionally, TBARS levels followed a similar decreasing trend, with the most pronounced effect in the PEOG group (0.45 ± 0.03 on day 3 to 0.25 ± 0.02 µmol/mL on day 10). The GB group exhibited modest reductions, but TBARS values remained significantly higher than PEOG throughout the study protocol. On the contrary, CTRL animals showed the highest TBARS levels compared to GB and PEOG groups across all time points, ranging from 0.81 ± 0.05 on day 3 to 0.72 ± 0.04 µmol/mL on day 10, indicating sustained oxidative damage in untreated animals (Figure 5C). Moreover, the PEOG group showed continuous reduction in values from 2.99 ± 0.14 on day 3 to 2.52 ± 0.12 on day 10 in NO_2_^−^, indicating the most effective decrease among groups. In contrast, both GB and CTRL groups maintained higher levels, with statistically significant differences observed on day 6 and day 10 compared to PEOG group, suggesting limited antioxidant effect in the absence of *P. erecta* extract (Figure 5D).

**Figure 6:**
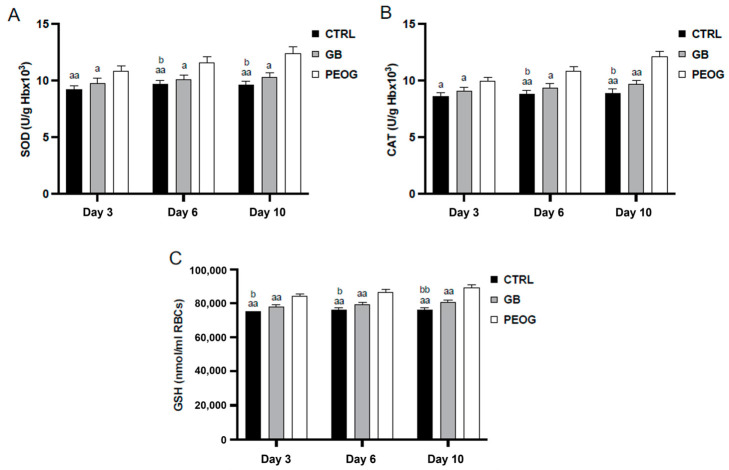
Values of antioxidative parameters in the plasma samples. (**A**)—superoxide dismutase (SOD), (**B**)—catalase (CAT); (**C**)—glutathione (GSH). CTRL—control, untreated rats; GB—rats treated with gel base; PEOG—rats treated with *P. erecta* oral gel. a—compared to PEOG; b—compared to GB; a and b—*p* < 0.05; aa and bb—*p* < 0.01.

Regarding the markers of antioxidative defense system, SOD activity was highest in the PEOG group, showing a consistent increase from 10.89 ± 0.4 U/g Hb × 10^3^ on day 3 to 12.46 ± 0.52 U/g Hb × 10^3^ on day 10, indicating a strong stimulation of antioxidant defense. In comparison, both the GB and CTRL groups exhibited significantly lower SOD activity at all time points compared to PEOG, with only modest changes over time. Additionally, the CTRL group showed significantly lower SOD activity than GB on days 6 and 10, suggesting impaired endogenous antioxidant response in untreated animals (Figure 6A). CAT activity followed a similar pattern, with the PEOG group showing a steady increase from 9.97 ± 0.35 U/g Hb × 10^3^ on day 3 to 12.14 ± 0.47 U/g Hb × 10^3^ on day 10. Both GB and CTRL groups displayed significantly lower values compared to PEOG at all time points. Additionally, CAT activity in the CTRL group was significantly reduced compared to GB on days 6 and 10 (Figure 6B). Moreover, GSH levels were also highest in the PEOG group, reflecting a sustained stimulation of non-enzymatic antioxidant defense. In contrast, GB and CTRL groups exhibited markedly lower GSH concentrations throughout the study, with the CTRL group showing significantly lower values than GB at all time points, further supporting the compromised antioxidant capacity in untreated animals with acetic-acid induced oral ulcer (Figure 6C).

The observed reduction in pro-oxidative markers and enhancement of antioxidant defense mechanisms in the PEOG-treated group may have played a crucial role in accelerating the healing of oral ulcerations. Oxidative stress is recognized as one of the key pathophysiological factors involved in the development and persistence of oral mucosal lesions. Excessive production of ROS, including O_2_^−^ and H_2_O_2_, can lead to lipid peroxidation, protein oxidation, DNA damage, and prolonged inflammatory responses, ultimately delaying tissue repair and re-epithelialization. Elevated TBARS levels observed in untreated animals further confirm the presence of enhanced lipid peroxidation and oxidative tissue damage within ulcerated mucosa. Simultaneously, the increased activities of SOD and CAT, together with elevated GSH concentrations, indicate a restoration of endogenous antioxidant defense systems.

The beneficial antioxidant effects of PEOG are likely associated with the high content of polyphenolic compounds present in *Potentilla erecta* extract. Polyphenols are well known for their ability to directly scavenge free radicals, chelate transition metals, and modulate cellular antioxidant pathways. Previous studies have demonstrated that tannins, flavonoids, and phenolic acids present in *P. erecta* exhibit potent antioxidant and anti-inflammatory properties, which may contribute to improved wound-healing outcomes and reduced tissue damage. Furthermore, the reduction in oxidative stress may indirectly suppress the activation of pro-inflammatory signaling pathways, creating a microenvironment more favorable for mucosal regeneration [17,34,35]. Therefore, the pronounced antioxidant activity observed in the PEOG group may represent one of the principal mechanisms underlying its therapeutic efficacy in acetic acid-induced oral ulceration.

The therapeutic effects observed with the *P. erecta* gel are consistent with previous reports describing the beneficial effects of topical formulations used for oral ulcer management. Hyaluronic acid-based gels, which are widely employed as protective and wound-healing agents in recurrent aphthous ulceration, have been reported to reduce pain intensity, decrease ulcer size, and accelerate healing. Similarly, topical corticosteroids such as triamcinolone acetonide remain among the most frequently used treatments owing to their anti-inflammatory activity. Although direct comparison is limited by differences in study design, ulcer models, treatment regimens, and outcome measures, the healing-promoting effects observed in the present study suggest that the *Potentilla erecta* gel may have therapeutic potential comparable to other locally applied formulations used for oral ulcer management [36,37].

## 3. Conclusions

Our findings, for the first time, suggest the therapeutic potential of a mucoadhesive gel based on *Potentilla erecta* extract in promoting oral ulcer healing. The beneficial effects of this formulation were evidenced by a marked increase in the percentage of ulcer contraction throughout the treatment period. Furthermore, 10 days of treatment with *P. erecta* gel significantly reduced pro-oxidant generation in buccal tissue, indicating attenuation of oxidative stress as a key mechanism contributing to its wound-healing activity. These findings are in agreement with previous reports indicating that successful treatment of oral ulcerations is often associated with reduced oxidative stress and improved antioxidant status.

These findings indicate that PEOG possesses considerable ulcer-healing potential and may represent a promising mucoadhesive formulation for the treatment of oral ulcerations.

## 4. Materials and Methods

### 4.1. Plant Material

The dried rhizome of the plant *P. erecta*, used in this study, was obtained from the Institute for Medicinal Plants Research “Dr. Josif Pancic”, Belgrade, Serbia. The dried rhizome was finely chopped, and then an ethanolic extract was made via ultrasonic extraction, with 70% ethanol solution as a solvent. In order to obtain a solid extract, liquid extract solution was converted to solid via low-pressure evaporation (RV05 basic IKA, IKA^®^ Werke GmbH&Co., Staufen im Breisgau, Germany), and then used to prepare a semi-solid formulation.

### 4.2. Formulation of P. erecta Extract-Based Gel

The gel base consisted of poloxamer 407 (Kolliphor^®^ P 407, pharmaceutical grade, BASF, Ludwigshafen, Germany), carbomer 934 (Carbopol^®^ 974P NF, NF grade, Lubrizol, Farmsum, The Netherlands), propylene glycol (pharmaceutical grade, Comcen d.o.o., Belgrade, Serbia), triethanolamine (reagent grade, ≥98%, Sigma Aldrich, St. Louis, MO, USA) and purified water (Ph. Eur.). For complete swelling, the carbomer dispersion was kept at rest for 24 h, and then poloxamer 407 was dispersed in the carbomer dispersion and left to stand for 24 h at 4 °C. Then, a triethanolamine solution was added to adjust pH of gel from 5.0 to 5.5. Finally, the *P. erecta* extract was slowly added with continuous mixing until a homogeneous hydrogel was obtained. Mixing was carried out using a laboratory mixer with a digital propeller (IKA^®^-Werke GmbH & Co. KG, Staufen, Germany) and a speed of 400 rpm [38]. The *P. erecta* ethanolic extract used in this study was characterized and described previously with defined chemical composition [14].

### 4.3. Assessment of Stability of PEG Formulation

#### 4.3.1. Organoleptic Characteristics and Physical Appearance

Changes in the colour, odour, consistency, and homogeneity of the formulated gel were assessed during three months of storage under different temperature conditions, 24 h after preparation and after 90 days of storage.

#### 4.3.2. Determination of pH Values

The pH of the formulated gel was measured using a calibrated digital pH meter (Mettler Toledo, Columbus, OH, USA). Prior to analysis, the instrument was calibrated with standard buffer solutions of pH 4.0, 7.0, and 9.0. All pH measurements were performed in triplicate to ensure accuracy and reproducibility [39].

#### 4.3.3. Determination of the Electrical Conductivity

The electrical conductivity of the prepared gel was measured using a CON 700 conductivity meter (Eutech Instruments, Thermo Fisher Scientific, Shanghai, China). Measurements were carried out in triplicate at room temperature with a stabilization time of 2 min [40].

#### 4.3.4. Centrifugation Test

For the centrifugation test, 10 g of the prepared gel was transferred into a conical test tube and centrifuged twice at 3000 rpm for 15 min at room temperature using a Hettich Mikro 120 centrifuge (Kirchlengern, Germany). Following centrifugation, the formulation was visually examined for signs of instability, including sedimentation and phase separation [41].

#### 4.3.5. Rheological Characterization

Rheological characterization of PEG was carried out using an Anton Paar MCR 102e rheometer (Anton Paar, Graz, Austria) fitted with a Peltier temperature control unit. Measurements were performed at 25 ± 0.1 °C and 37 ± 0.1 °C using a PP25 plate–plate geometry with a 1 mm gap. Samples were equilibrated for 3 min before testing. RheoCompass software (version 1.31; Anton Paar, Graz, Austria) was used for instrument control and data acquisition [42].

#### 4.3.6. Swelling Index

To assess the swelling capacity, 1 g of gel was placed in a Petri dish containing 5 mL phosphate buffer (pH 5.5). The samples were incubated and weighed after 1 h and 3 h to determine the extent of swelling. The swelling index was calculated from the measured weight gain using the appropriate equation. The same experimental procedure was performed after 90 days of storage for samples kept at 4 ± 2 °C and 25 ± 2 °C to evaluate the effect of storage conditions on swelling behavior [43].

Swelling Index (SW)% = [Wt − Wo/Wo] × 100

(SW)% = Equilibrium percent swelling

Wt = Weight of swollen gel after time t

Wo = Initial weight of gel

### 4.4. Determination of Antioxidative Activity

#### 4.4.1. Total Phenolic Content

The total phenolic content of the investigated extract was determined spectrophotometrically using the Folin–Ciocalteu reagent with slight modifications of a previously described procedure [42]. Briefly, 100 µL of the methanolic extract solution was mixed with 0.75 mL of diluted Folin–Ciocalteu reagent and incubated for 5 min at room temperature. Subsequently, 0.75 mL of sodium bicarbonate solution (60 g/L) was added to the reaction mixture. After 90 min of incubation under ambient conditions, absorbance was measured at 725 nm. Gallic acid was used for calibration curve preparation, and the results were expressed as mg gallic acid equivalents per gram of dry extract (mg GAE/g dry extract). All analyses were performed in triplicate, and data are presented as mean ± standard deviation (SD).

#### 4.4.2. DPPH Radical-Scavenging Activity

The antioxidant activity of the extract was evaluated using the DPPH radical-scavenging assay adapted to a 96-well microplate format according to previously reported methodology with minor modifications [44]. A freshly prepared methanolic DPPH solution (0.004%, *w*/*v*) was mixed with appropriate concentrations of the tested samples and standard antioxidants to obtain a final reaction volume of 200 µL per well. The mixtures were incubated in the dark at room temperature for 30 min, after which absorbance was recorded at 515 nm. Radical-scavenging activity was expressed as IC_50_ values, representing the concentration required to inhibit 50% of DPPH radicals. Ascorbic acid, BHA, and Trolox were used as reference antioxidants. All measurements were carried out in triplicate.

#### 4.4.3. ABTS Radical-Scavenging Assay

The ABTS radical cation decolorization assay was used to further evaluate the antioxidant potential of the investigated extract following a previously published protocol with slight modifications [44]. The ABTS^•+^ working solution was prepared by reacting ABTS stock solution with potassium persulfate and allowing the mixture to stand in the dark for 16 h before use. Prior to analysis, the solution was diluted with ethanol to achieve an absorbance of 0.700 ± 0.020 at 734 nm. For the assay, 20 µL of the tested sample or standard antioxidant solution was mixed with 180 µL of ABTS^•+^ reagent in 96-well plates. The decrease in absorbance was measured at 734 nm after 1 min of reaction. Antioxidant activity was expressed as IC_50_ values. All experiments were performed in triplicate.

#### 4.4.4. Ferric-Reducing Antioxidant Power Assay

The ferric-reducing antioxidant power (FRAP) assay was employed to assess the reducing capacity of the extract according to a previously described method with minor modifications [41]. The FRAP working reagent was freshly prepared by mixing acetate buffer (300 mM, pH 3.6), TPTZ solution (10 mM in 40 mM HCl), and ferric chloride solution (20 mM) in a 10:1:1 ratio. Subsequently, 10 µL of the tested sample was added to 300 µL of FRAP reagent and incubated at 37 °C for 10 min. Absorbance was measured at 593 nm using a microplate reader. Trolox was used as a positive control, whereas ferrous sulfate solutions served for calibration curve construction. The obtained antioxidant capacity values were expressed as µmol Fe^2+^ equivalents. All measurements were performed in triplicate.

### 4.5. In Vivo Oral Ulcer-Healing Examinations

Specimens used in this study were laboratory rats (Wistar albino), 60 male rats, 8–10 weeks old, body weight 200–250 g, acquired from the Military Medical Academy, Belgrade, Serbia. All animals were kept in strictly controlled vivarium conditions (temperature 22 ± 1 °C, light–dark cycle 12:12 h) and relative humidity 55–60%. To minimize the potential influence of mastication and swallowing on formulation retention, food was withheld for 60 min following gel administration, whereas water remained available ad libitum. After this period, the animals were returned to their normal feeding regimen. The complete experimental procedure was approved by the Ethical Committee for the Protection of the Welfare of Experimental Animals of the Faculty of Medical Sciences (No. 09–12630/2). The research was conducted in the Center for Preclinical and Functional Research, Faculty of Medical Sciences, University of Kragujevac, Serbia. All experimental procedures were in accordance with EU Directive 86/609/EEC and the principles of good laboratory practice (GLP).

#### 4.5.1. Induction of the Oral Buccal Lesion

To induce the oral buccal ulcer model, rats were first anesthetized with a mixture of ketamine (5 mg/kg) and xylazine (10 mg/kg) intraperitoneally. An ulcer on the oral mucosa was formed by exposing the buccal surface to glacial acetic acid according to a previously established in vivo model [44]. Chronic ulceration developed two days later with clearly defined borders, which was designated as day 0.

Following the induction of oral ulceration, treatment was initiated immediately by topical administration of the assigned formulation once per day. The animals were randomly allocated into three experimental groups (n = 20 per group): untreated control, gel base (GB), and PEOG-treated.

The formulations were applied directly to the ulcerated area using a cotton swab mounted on a plastic applicator. A dose of 0.5 g of gel was administered once per day until healing was achieved. To evaluate the healing process over time, five animals from each group were euthanized on days 0, 3, 6, and 10 after ulcer induction. Following brief anesthesia with ketamine/xylazine, the animals were sacrificed by decapitation. Blood samples were collected for the assessment of systemic oxidative stress parameters, while buccal tissue specimens were harvested for histopathological examination and analysis of local redox status.

#### 4.5.2. Assessment of Oral Buccal Lesion Healing

The assessment of oral buccal lesion healing was monitored daily from day zero until complete healing, while the size of the ulceration was determined using Image J software (version 1.53; National Institutes of Health, Bethesda, MD, USA). The degree of healing was expressed as a percentage and was calculated for each animal according to the following formula:

Ulcer healing rate (%) = (A_0_ − At) × 100/A_0_,

where A_0_ represents the initial ulcer area measured on day zero, and At is the ulcer area at the time of observation.

### 4.6. Histochemical Analysis

Samples of aphthous lesions were fixed in 4% formalin for 24 h. After fixation, the tissues were dehydrated in a series of rising alcohol concentrations (70%, 96% 100%), cleaned in xylene and embedded in paraffin. Hematoxylin and eosin (H/E) staining was applied to thin slices (5 μm) in order to assess changes in tissue morphology. An Olympus BX51 microscope with a digital camera was used to take microscopic pictures.

The samples were examined using the technique outlined by Vuletić et al. [38].

The histological scoring procedure used was as follows:

1. Presence of epithelial necrosis, with no signs of inflammation.
2. Onset of the inflammatory response, without new capillary proliferation.
3. Prominent inflammatory reaction with limited capillary proliferation at the base of the ulcer, but no epithelialization at the surface.
4. Reduced inflammation, newly formed capillaries reaching the surface, and initiation of epithelialization.
5. Complete epithelialization.

### 4.7. Biochemical Analysis

After sacrificing the animals, blood samples were collected from the jugular vein for biochemical parameters. The following oxidative stress parameters were determined from the plasma sample: superoxide anion radical (O_2_^.−^), hydrogen peroxide (H_2_O_2_), nitric oxide, measured as nitrite (NO^2−^)^,^ and lipid peroxidation index (TBARS). The following antioxidant protection parameters were determined from the erythrocyte lysate: superoxide dismutase (SOD), reduced glutathione (GSH), and catalase (CAT).

#### 4.7.1. Determination of Prooxidants in Plasma Samples

The determination of superoxide anion radicals (O^2.−^) is based on the reaction of superoxide anion radicals with nitro-tetrazolium blue. The test protocol involves the use of TRIS reagent, Na_2_EDTA, gelatin and nitro-tetrazolium blue chloride. The absorbance was measured spectrophotometrically at a wavelength of λ = 550 nm.

The determination of hydrogen peroxide is based on the oxidation of phenol red, which in the presence of H_2_O_2_ is catalyzed by the enzyme peroxidase from horseradish. The absorbance was measured spectrophotometrically at a wavelength of λ = 610 nm.

The determination of nitrites is based on the use of Griess reagent, which in the presence of nitrite leads to the formation of a diazo complex. The level of nitrite actually represents the level of liberated nitric oxide. The absorbance at the wavelength λ = 550 nm was measured spectrophotometrically.

The determination of the index of lipid peroxidation (TBARS) is performed indirectly via the reaction products of lipid peroxidation with thiobarbituric acid. The test protocol involves the use of 1% TCA (thiobarbituric acid) and NaOH. The absorbance at the wavelength λ = 530 nm was measured spectrophotometrically [45].

#### 4.7.2. Determination of Antioxidant Values in Erythrocyte Samples

The determination of superoxide dismutase is performed by the epinephrine method. A total of 100 μL of epinephrine is added to a mixture of 100 μL of lysate and 1 mL of carbonate buffer. The absorbance is measured at a wavelength of λ = 470 nm.

The determination of the level of reduced glutathione is performed based on GSH oxidation with 5,5-dithio-bis-6,2-nitrobenzoic acid. The absorbance is measured at a wavelength of λ = 420 nm.

Catalase determination involves the use of lysate diluted with distilled water (1:7 *v*/*v*) and treated with chloroform–ethanol (0.6:1 *v*/*v*), followed by 50 μL of CAT buffer, 100 μL of prepared sample and 1 mL of 10 mM H_2_O_2_. Absorbance was measured at a wavelength of λ = 360 nm [45].

### 4.8. Statistical Analysis

All data were analyzed using GraphPad Prism 8 (Version for Windows, GraphPad Software, La Jolla, CA, USA). The results are expressed as means ± standard deviation of the mean (SD) or percentages, depending on the data type. The distribution of the data was checked by the Shapiro–Wilk test. An independent samples *t*-test (parametric) and a Mann–Whitney U test (nonparametric), as well as a one-way ANOVA and a Kruskal–Wallis test, were used to assess the difference in estimated variables between the groups. A *p*-value < 0.05 was regarded as statistically significant.

## Figures and Tables

**Table 1:** Sensory and physical stability of PEOG during 3-month storage at 25 °C and 4 °C.

Parameter	25 ± 2 °C (Day 1)	25 ± 2 °C (Day 90)	4 ± 2 °C (Day 1)	4 ± 2 °C (Day 90)
Color	Light brown	Light brown	Brownish	Brownish
Odor	Distinct extract odor	Distinct extract odor	Distinct extract odor	Distinct extract odor
Consistency	Semi-solid	Semi-solid	Semi-solid	Semi-solid
Homogeneity	Uniform, no separation	Uniform, no separation	Uniform, no separation	Uniform, no separation

**Table 2:** pH changes in PEOG gel during 3-month storage at 25 °C and 4 °C.

Storage Temperature	pH (Day 1)	pH (Day 90)
25 ± 2 °C	5.75 ± 0.11	5.91 ± 0.14
4 ± 2 °C	5.79 ± 0.12	5.88 ± 0.16

Data are presented as mean ± SD (n = 3).

**Table 3:** Electrical Conductivity changes in PEOG during 3-month storage at 25 °C and 4 °C.

Storage Duration	25 ± 2 °C (µS/cm)	4 ± 2 °C (µS/cm)
Day 1	50.7 ± 0.50	50.7 ± 0.50
Day 90	46.1 ± 0.58 *	68.3 ± 0.60 *#

Data are presented as mean ± SD (n = 3); *—statistical significance in relation to day 1; #—statistical significance in relation to 25° ± 2 °C.

**Table 4:** Swelling index of PEOG immediately after preparation and after 90 days of storage at 4 ± 2 °C and 25 ± 2 °C.

Samples/Hours	0 h (Day 1)	1 h (Day 1)	3 h (Day 1)	0 h (Day 90)	1 h (Day 90)	3 h (Day 90)
PEOG	88 ± 0.17	94 ± 0.15	133 ± 0.12	–	–	–
PEOG 4 ± 2 °C	–	–	–	82 ± 0.18	77 ± 0.14	72 ± 0.17
PEOG 25 ± 2 °C	–	–	–	80 ± 0.23	93 ± 0.16	110 ± 0.20

**Table 5:** IC_50_ values of antioxidant activity of the tested samples determined by DPPH and ABTS assays.

Samples	DPPH IC_50_ (µg/mL)	ABTS IC_50_ (µg/mL)
Extract	58.94 ± 5.09 ^a^	68.67 ± 7.04 ^a^
AA	7.41 ± 0.63 ^b^	8.47 ± 0.74 ^b^
BHA	9.86 ± 0.84 ^b^	11.54 ± 0.95 ^b^
Trolox	4.29 ± 0.13 ^b^	5.58 ± 0.61 ^b^

Data are expressed as mean ± SD (n = 3). Values marked with different superscript letters within the same column differ significantly according to one-way ANOVA followed by Tukey’s multiple comparison test (*p* < 0.001). Lower IC_50_ values correspond to stronger antioxidant activity. DPPH—2,2-diphenyl-1-picrylhydrazyl radical-scavenging assay; ABTS—2,2′-azinobis (3-ethylbenzothiazoline-6-sulfonic acid) radical cation scavenging assay; AA—ascorbic acid; BHA—butylated hydroxyanisole; Trolox—water-soluble analogue of vitamin E (6-hydroxy-2,5,7,8-tetramethylchroman-2-carboxylic acid).

**Table 6:** Histological scoring results for buccal ulcer tissues.

	0 Day	3 Day	6 Day	10 Day
CTRL	2.00 ± 0.00	2.83 ± 0.41	3.83 ± 0.41	4.83 ± 0.41
GB	2.33 ± 0.52	3.33 ± 0.52	4.33 ± 0.52	5 ± 0.00
PEOG	2.67 ± 0.52	4 ± 0.00	5 ± 0.00	/

CTRL—control, untreated rats; GB—rats treated with gel base; PEOG—rats treated with *P. erecta* oral gel.

## Data Availability

The raw data supporting the conclusions of this article will be made available by the authors on request.

## Abbreviations

The following abbreviations are used in this manuscript:

PEOG	*Potentilla erecta* L. ethanol extract
AA	Ascorbic acid
ABTS	2,2′-azinobis(3-ethylbenzothiazoline-6-sulfonic acid) radical cation scavenging assay
BHA	Butylated hydroxyanisole
CAT	Catalase
CTRL	Control
DPPH	2,2-diphenyl-1-picrylhydrazyl radical-scavenging assay
FRAP	Ferric-reducing antioxidant power
GB	Gel base
GLP	Good laboratory practice
GSH	Reduced glutathione
H/E	Hematoxylin and eosin
H_2_O_2_	Hydrogen peroxide
NO^2−^	Nitrites
SD	Standard deviation
SOD	Superoxide dismutase
SW	Swelling Index
TBARS	Lipid peroxidation index
TCA	Thiobarbituric acid
TPC	Total phenolic content
O_2_^.−^	Superoxide anion radical
